# Nanobodies—Useful Tools for Allergy Treatment?

**DOI:** 10.3389/fimmu.2020.576255

**Published:** 2020-09-30

**Authors:** Sabine Flicker, Ines Zettl, Sergei V. Tillib

**Affiliations:** ^1^ Division of Immunopathology, Institute of Pathophysiology and Allergy Research, Center for Pathophysiology, Infectiology and Immunology, Medical University of Vienna, Vienna, Austria; ^2^ Institute of Gene Biology, Russian Academy of Sciences, Moscow, Russia

**Keywords:** allergy, allergen, nanobody, V_H_H, blocking antibody, allergy treatment

## Abstract

In the last decade single domain antibodies (nanobodies, V_H_H) qualified through their unique characteristics have emerged as accepted and even advantageous alternative to conventional antibodies and have shown great potential as diagnostic and therapeutic tools. Currently nanobodies find their main medical application area in the fields of oncology and neurodegenerative diseases. According to late-breaking information, nanobodies specific for coronavirus spikes have been generated these days to test their suitability as useful therapeutics for future outbreaks. Their superior properties such as chemical stability, high affinity to a broad spectrum of epitopes, low immunogenicity, ease of their generation, selection and production proved nanobodies also to be remarkable to investigate their efficacy for passive treatment of type I allergy, an exaggerated immune reaction to foreign antigens with increasing global prevalence.

## Introduction

Type I allergy, an IgE antibody mediated hypersensitivity disease, represents a common health problem affecting almost 30% of the population worldwide (1). The recognition of allergens by specific IgE antibodies that are generated after sensitization is a key event for the initiation of allergic inflammation (2). Allergic patients suffer from a variety of allergic symptoms including rhinoconjunctivitis and asthma (3) but also food allergy and skin inflammation (4). These clinical manifestations cause a major burden by reducing the quality of life of affected persons (5). While anti-inflammatory treatment based on pharmacotherapy reduces allergic symptoms and is the most commonly prescribed medication for treatment of allergic patients (6), only allergen-specific immunotherapy (AIT) represents a causative treatment of type I allergy. In fact, AIT induces a protective immunity in allergic patients based on the modification of cellular and humoral responses to the disease causing allergen (7). Besides the inhibition of IgE binding to their specific allergen, the immune deviation from a TH2 to TH1 response, and the decreases in numbers of effector cells in target organs, the generation and maintenance of allergen-specific regulatory T and B cells and the involvement of their suppressive cytokines are essential for the induction of allergen tolerance (8–10). Beyond doubt the improvement of allergic symptoms is further caused by AIT-induced IgG antibodies found in serum and nasal secretions (8, 11–14). For many years AIT was conducted with aqueous natural allergen extracts and patients experienced considerable side effects due to the unpredictable composition and poor quality of the injected extracts (1). Recent developments like next-generation forms of AIT based on molecular approaches may overcome the limitations of current forms of AIT (15, 16). The last generation of improved vaccines, i.e. peptide carrier vaccines, induces an IgG response that targets IgE binding sites on allergens. Induced IgG antibodies effectively block IgE binding and are termed blocking antibodies (1, 17).

However, the efficacy of such blocking antibodies was long questioned because it revealed to be cumbersome to isolate reproducible defined, i.e. monoclonal allergen-specific antibodies comprising the capacity to inhibit allergen-induced allergic reactions.

A recent proof of concept study re-stimulated the idea to generate monoclonal allergen-specific antibodies and to evaluate their feasibility for allergy treatment. The authors could show that a single subcutaneous injection of a mixture of two human monoclonal allergen-specific IgG4 antibodies significantly reduced allergic symptoms in allergic patients (18, 19). Moreover, validated in a PCA mouse model, the mixture of these two monoclonal antibodies proved to be more potent in inhibiting mast cell degranulation than IgG antibodies purified from patients’ sera who underwent successful AIT (18). Furthermore, these human monoclonal IgG4 antibodies recently completed the phase II clinical trial in treatment of cat allergic patients (https://clinicaltrials.gov/ct2/show/NCT03838731). These results proved for the first time that allergy treatment with monoclonal allergen-specific antibodies is a well-tolerated, rapid, and effective approach to reduce allergic inflammation and rekindled the blocking antibody concept (11, 20, 21).

Nevertheless, the generation and identification of blocking conventional human or humanized antibodies is connected with high costs for production, validation and application (22, 23). Therefore, cost-effective alternatives are currently sought.

The nanobody technology represents such an alternative implying a significant improvement to the laborious methods to obtain monoclonal blocking conventional antibodies. Due to their beneficial properties of small molecules and monoclonal antibodies, nanobodies in general are an attractive agent for development of novel therapeutic strategies (24, 25). The ease of their generation and production, the single domain organization, their beneficial biochemical properties and their feature to recognize small cavities on the surface of antigens and hence bind to epitopes inaccessible for conventional antibodies (26) have raised the particular interest of allergologists recently.

Can the nanobody technology provide enhanced opportunity to generate a panel of antigen-binding molecules with various epitope specificities for certain allergens different to conventional antibodies? Will these identified allergen-specific nanobodies be more efficient in blocking than conventional IgG antibodies due to their pronounced cleft recognition? Will it be possible with this technology to find single nanobodies that are able to abrogate IgE-mediated allergic inflammation? These questions and our wish to answer these questions attracted our attention. Within this review, we focus on the powerful nanobody technology to generate allergen-specific nanobodies and report on their evaluation for prospective application for passive allergy treatment.

## The Complex and Laborious Approach to Identify Effective, Protective Allergen-Specific Monoclonal Antibodies

If allergologists are asked why the search for effective protective allergen-specific monoclonal antibodies is complex and laborious, they will describe this issue by the typical quest for a needle in a haystack. Through intense and precise molecular and immunological exploration of available allergen-specific monoclonal antibodies in the past it was proven that epitope specificity and affinity are decisive for their inhibitory potential to block IgE binding and thus IgE-mediated reactions (21, 27–29). The commitment to find and isolate monoclonal antibodies with specificity and high affinity for certain allergens and even more for certain epitopes always started with several fundamental decisions. Amongst them the choice for the perfect source to gain DNA coding for antibodies and the applied technology to generate allergen-specific antibodies are two of the most critical ones. Regarding the DNA source both animals, mainly mice, and humans served as blood, spleen, tonsils and even bone marrow donors in the last decades to isolate B cells or plasma cells and thus DNA coding for antibodies (30–32). For the proof of principle, murine IgG antibodies overlapping with human IgE binding sites are valuable tools to investigate the effects to inhibit IgE epitope recognition on allergens and consequently to contribute to the design of hypoallergenic derivatives suitable for AIT (33). However, the direct therapeutic use of these murine monoclones in humans is limited by the high incidence of harmful immune responses against these administered foreign proteins (34). To mitigate this limitation numerous murine monoclonal antibodies have been re-engineered by chimerization and humanization technologies. These expensive procedures are justified for fatal diseases like different forms of cancer but were barely applied for allergen-specific murine antibodies so far with a few exceptions (35, 36). This was one of the main reasons why allergologists in the recent past endeavour to focus on human donors including allergic patients, AIT-treated patients and even healthy individuals depending on the research question (28, 37, 38).

Various methods were utilized to generate allergen-specific genuine, i.e. native antibodies with the preservation of the natural VH and VL pairing including hybridoma technology, Epstein-Barr-Virus (EBV) transformation, single B cell sorting and cloning and HumAb mice (transgenic mice that produce fully human antibodies) (18, 39–49). In parallel, versatile approaches were developed to generate non-genuine antibodies by random combination of VH and VL chains, i.e., combinatorial Fab/ScFv libraries or (semi-) synthetic libraries (37, 38, 50–60). Based on PCR amplification as strong tool to depict large antibody repertoires and phage display to screen these large repertoires, many recombinant allergen-specific antibody fragments (Fabs or ScFvs) were isolated (37, 38, 50–56, 58–64).

All mentioned technologies have definitely contributed to the isolation and evaluation of monoclonal allergen-specific IgG, IgE antibodies and fragments thereof and furthermore to assess their feasibility for allergy treatment. Nevertheless, all mentioned technologies are also reported to have some limitations. While the hybridoma technology and EBV transformation are generally unsuitable for a comprehensive screening of large antibody repertoires because of their inefficient fusion and transformation events, the single B cell sorting was long hampered by inadequate staining technologies to clearly identify allergen-specific antibody producing cells (32, 39). The main drawback of combinatorial libraries is that they usually rely on random combination and thus most likely unnatural VH and VL antibody pairings. Additionally, it turned out independent of the applied technology to be very difficult to isolate monoclonal IgG and IgE antibodies with a broad epitope spectrum for each allergen. It also revealed that besides several blocking antibodies also many non-blocking or even enhancing antibodies were isolated (44, 63–65). While all three types of monoclonal antibodies were unambiguously supportive to study the structural requirements for efficient effector cell activation and hence contribute to elucidate the underlying mechanisms of type I allergy, non-blocking and enhancing antibodies were fully useless for the prospective application as protective antibodies.

These insights forced allergologists to look beyond the conventional antibody horizon.

## The Powerful Nanobody Technology to Isolate Allergen-Specific Nanobodies—A Welcome Alternative to Conventional Antibody Generation

About 30 years ago, a group of Belgian scientists made an unexpected discovery, which was patented and later presented to the scientific community in the form of the well-known discovery publication in the journal Nature in 1993 (66). They found that a significant amount of non-canonical types of antibodies is naturally present in blood of Camelidae in addition to conventional antibodies. This exceptional type of antibody called Heavy Chain-only Antibody (HCAb) lacks light chains and consists of a homodimer of shortened (without CH1 domain) heavy chains. The antigen-recognition region in HCAbs is formed by only one variable domain (V_H_H) that is directly linked *via* a hinge region to the Fc-domain (66). Later on, similar non-canonical HCAbs were found in some cartilaginous fishes such as sharks and ratfish (67–69). The antigen-binding variable domain of these antibodies was named VNAR as opposed to V_H_H in camelids. A recombinant protein version of the V_H_H-or VNAR-domain is usually called “single domain antibody” or “nanobody”. The very popular term “nanobody” is the commercial name given by the Belgian biopharmaceutical company Ablynx, a pioneer in HCAb-based therapeutic applications that was acquired by Sanofi in 2018.

The nanobody generation technology was proven to be a very efficient machinery to generate nanobodies with required properties and offered crucial advantages compared to traditional techniques utilized to produce murine or human conventional antibodies. After the typical initial immunization (of camelids) step, the full repertoire of cDNA coding for functional nanobodies can be efficiently cloned from peripheral blood lymphocytes of immunized animals using PCR amplification and then a panel of nanobodies of required specificity can be easily selected using phage (or other type of) display-based methods (66, 70–72). In addition, there are different *in vitro* affinity maturation approaches to improve features of initially selected nanobodies (71, 73, 74). In some cases, especially if the antigen of interest is toxic, unstable, non-immunogenic or not available in sufficient quantity, other types of libraries (naive, semisynthetic or fully synthetic libraries) can be efficiently used instead of immune libraries for generation of nanobodies (75–79). Synthetic libraries can be made using special predesigned scaffolds such as humanized scaffolds optimized for intracellular stability (77) or optimized for bacterial expression (80). Non-immune libraries are typically much larger than immune libraries and a ribosome display was suggested for the initial selection round of such large libraries to work with higher concentrations of nanobody variants than in case of phage display (79, 80). Synthetic libraries combined with different selection procedures were successfully used to obtain conformationally selective nanobodies against G protein-coupled receptors (78), sybodies against very challenging targets such as the heterodimeric bacterial ABC exporterTM287/288 (81) or the intracellular KDEL receptor (82) to name a few examples from many others.

**Figure 1:**
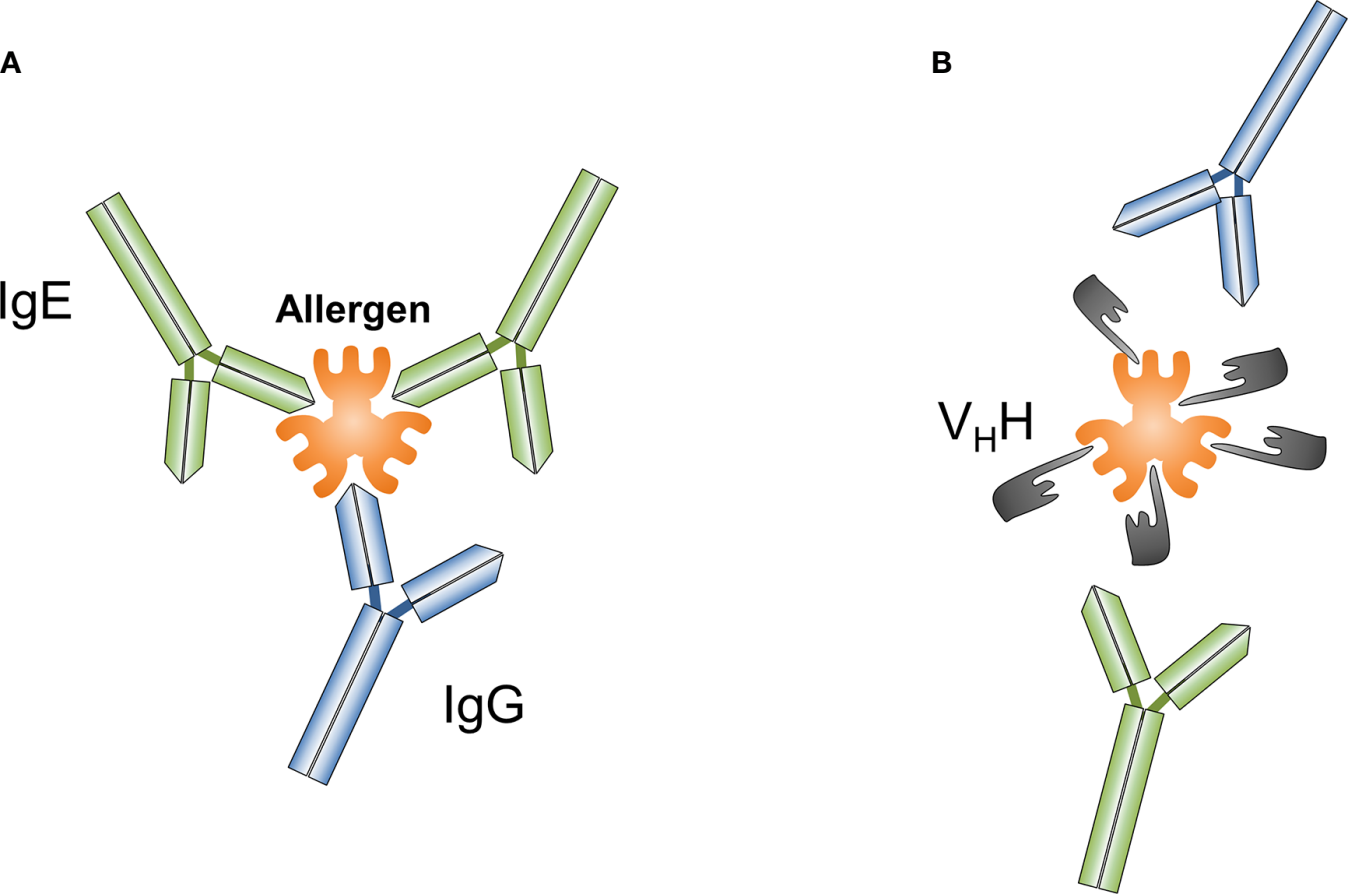
Conventional antibodies such as IgG or IgE **(A)** and nanobodies (V_H_H) **(B)** can be generated against different epitopes of targeted antigens, a particular allergen. Nanobodies overlapping with IgE binding sites on allergens prevent IgE-mediated allergic reactions.

Nanobodies comprise unique features that distinguish them from classical antibodies. Nanobodies are the smallest known antibody fragments (4 × 2.5 x 3 nm, 12–15 kDa) of natural origin that are able to specifically bind their cognate antigens. Due to their often extended CDR3 loop they can form unusual paratopes, i.e. finger-like extensions and thus recognize special native antigenic epitopes (small cavities, concave surfaces, conformational epitopes, active sites of enzymes) that are hidden for conventional antibodies (**Figure 1**). Indeed, nanobodies have proven to be useful tools for modulating the activity of enzymes (26, 83, 84). It could be therefore speculated that allergen-specific nanobodies that modulate or inhibit the proteolytic activity of certain allergens (*e.g.*, Phl p 1, Der p 1) might reduce their penetration capacity through mucosal surfaces. Furthermore, nanobodies are able to bind small peptides with high affinity (85–89). Their high affinity, solubility and stability over a wide range of temperatures and pH, ease of producing in bacteria or other expression systems make them convenient molecules for different applications, as well as for all possible engineering modifications *e.g.*, development of complex constructs and conjugates. Nanobody-based tools are therefore increasingly used for research, molecular visualization, diagnostics and development of new treatment options for various pathologies, including cancer and other socially significant diseases (71, 72, 90–94).

So far, only one allergen-specific nanobody is described in the literature. This nanobody is reported to be specific for the major peanut allergen, Ara h 3 and was isolated from a synthetic library of humanized nanobodies *via* phage display (95). The interaction between Ara h 3 and the Ara h 3-specific nanobody resulted in a dissociation constant of 400 nM representing medium affinity binding and was further investigated by the structural determination of formed co-crystals (95). The authors acknowledged that additional work is needed to improve the affinity of the isolated nanobody to make it an attractive tool for the development of biosensors for peanut allergen detection. This finding clarifies that the selection procedure is only one part of the successful discovery of potent IgE-blocking nanobodies, thus the evaluation of selected nanobodies is critical as well.

Nevertheless, we are confident that soon more allergen-specific nanobodies will arise to be studied for their potential to abrogate IgE-mediated allergic inflammation.

## Evaluation of the Suitability of Allergen-Specific Nanobodies for Allergy Treatment

**Figure 2:**
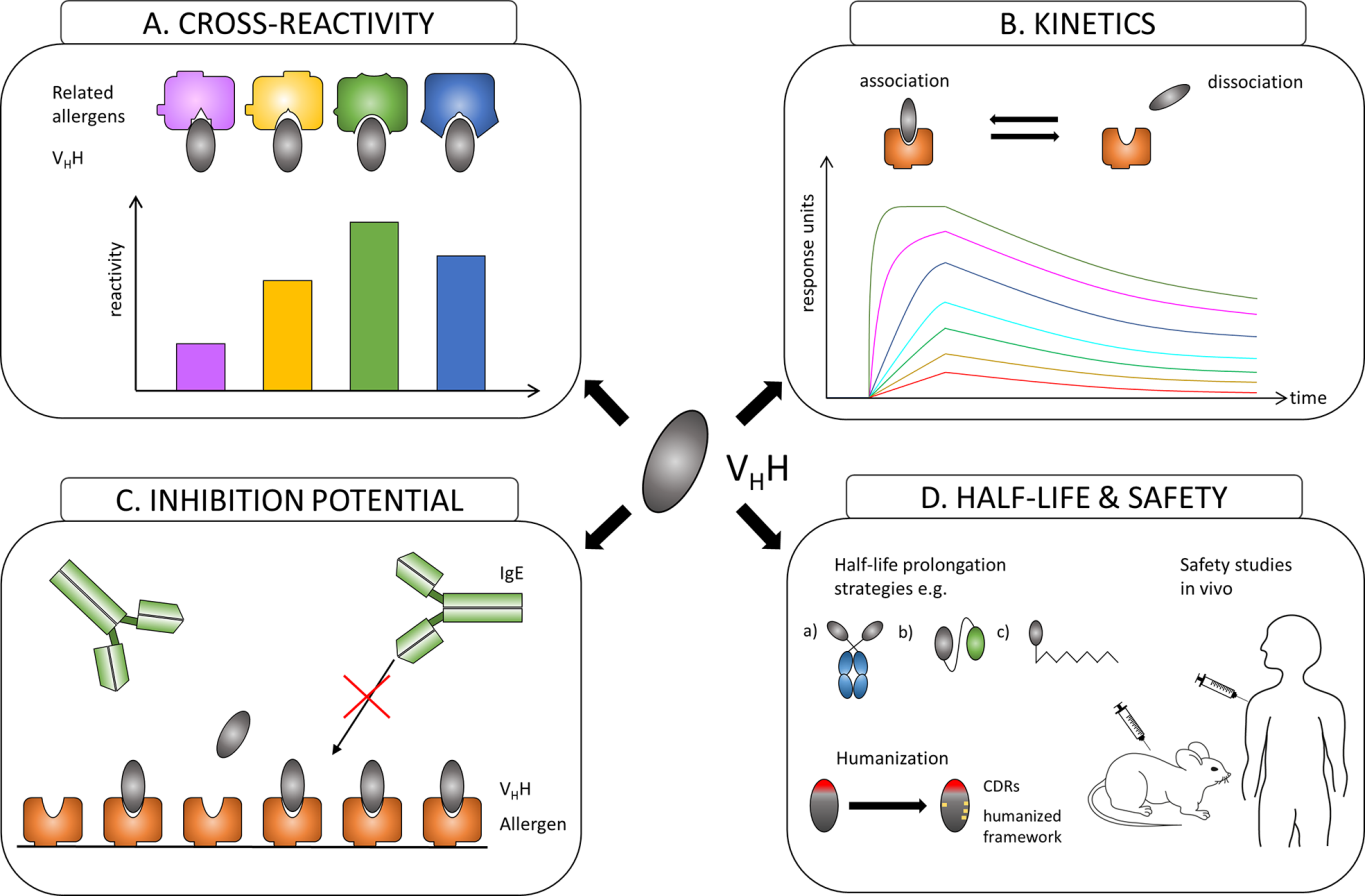
Overview of the evaluation process of the suitability of allergen-specific nanobodies for allergy treatment. **(A)** Evaluating cross-reactivity to related allergens. **(B)** Measuring affinities of selected candidates to the allergen. **(C)** Investigating the potential to block allergen-specific IgE from binding to the allergen. **(D)** Adjusting the half-life of a suitable nanobody by e.g.: a) linking to IgG Fc region; b) oligomerization to homomers or heteromers to facilitate linking to other proteins like human serum albumin (HSA); c) PEGylation. Increasing safety by humanization of the framework and performing safety studies *in vivo*.

Similar to the evaluation of conventional antibodies with the focus to identify effective protective monoclones, generated nanobodies have to be assessed first for their allergen specificity, epitope recognition, cross-reactivity to homologous allergens present in related species, for their affinity to their corresponding allergens and most importantly for their ability to inhibit patients´ IgE binding to these allergens (**Figures 2A–C**). After the allergen specificity of isolated nanobodies is confirmed, the proof for cross-reactivity (**Figure 2A**) is of great importance because IgE antibodies from allergic patients often displayed cross-reactivity to allergens from other allergen sources (28, 96). High affinity and slow dissociation of formed nanobody/allergen complexes will be critical prerequisites for allergen-specific nanobodies to be chosen as suitable candidate (**Figure 2B**). However, the pivotal characteristics for an allergen-specific nanobody to be attractive for further processing will be the determination of its potential to block patients’ IgE binding and hence IgE-mediated effector cell activation (**Figure 2C**). Additionally, specific nanobodies have to be tested as well for their cross-protectivity to homologous allergens. All these properties are crucial requirements for allergen-specific nanobodies to be selected for further essential investigations concerning half-life, clearance and safety.

Nanobodies are considered as proteins of weak immunogenicity due to the shared similarities with variable VH domains of human immunoglobulins (IgG3 subclass), and they can be further improved by a humanization approach (97) (**Figure 2D**). Consequently, no immune response against applied nanobodies was raised in mice or humans that were injected with nanobody-containing constructs (98–100). Safety of nanobody-based drugs is confirmed by several completed Phase 1 and Phase 2 clinical trials (101) and recent approval by the US Food and Drug Administration (FDA) and the European medicines agency (EMA) of the first therapeutic nanobody, Caplacizumab, a bivalent nanobody designed for the treatment of thrombotic thrombocytopenic purpura and thrombosis (102).

Though advantageous for *in vivo* imaging, the small size of nanobodies could be seen as a disadvantage for passive treatment of allergy due to a quick renal clearance of nanobodies from blood (approx. 30 min). Many different strategies to extend the *in vivo* half-life of nanobody-based construct have been developed (103). They include increasing the hydrodynamic radius of a protein by attaching highly flexible and hydrophilic molecules such as polyethylene glycol (PEG) and carbohydrates or by genetic fusion with polypeptide chains mimicking the biochemical properties of PEG, fusion of V_H_H to the Fc region of IgG, fusion or non-covalent binding to albumin (104) (**Figure 2D**). Nanobodies can also be used as modules to engineer larger molecules with several valencies and/or specificities, such as multivalent (105–108), bispecific (105, 109), and other (110, 111) constructs that may acquire considerably higher specificity, binding efficiency and biological activity (106, 107, 111). Nanobodies were also considered as possible ligands to design new highly specific immunosorbents (112–114).

Different types of nanobody-based tools/approaches can be envisaged to be potentially profitable for an allergy treatment: a) bispecific nanobodies for topical application to capture allergens before they penetrate epithelial mucosa in airways, b) very stable nanobodies to capture food allergen in gastrointestinal tract, c) anti-idiotypic nanobodies mimicking allergenic epitopes as a possible replacement for a complex natural allergen for a new kind of AIT vaccine development, d) multivalent nanobody-based constructs for systemical administration to efficiently block allergen interaction with IgE on mast or basophil cells, e) efficient immunosorbents to remove IgE from the blood by immune apheresis. Correspondingly, different administration approaches for nanobody-based constructs can be developed: aerosol or topical applications, oral route or subcutaneous administrations. Temporary blocking of allergen-IgE interaction (i.e. by topical or systemic administration of specific nanobodies) or a subtraction of IgE from the periphery blood (i.e. apheresis) may give a short-term treatment effect. For a long-term treatment effect we could hypothesize the use of anti-idiotypic nanobodies to IgE. Such nanobodies may represent “internal images” of an allergen and mimick hypoallergenic B cell epitopes. To efficiently induce IgG response that targets IgE binding sites on allergens, these nanobodies should be fused to a viral coat protein as it was described for next-generation forms of AIT (15).

## Conclusion and Perspective

The generation of allergen-specific nanobodies unambiguously represents a reasonable progress in the field of allergy. With their well-documented qualities including their ability to recognize unusual “hidden” epitopes, high affinity binding, solubility, extreme stability and low immunogenicity, nanobodies attracted the interest of allergologists to study their suitability for passive allergy treatment. The chance to find allergen-specific nanobodies with this powerful technology that ideally comprise high affinity and bind to epitopes partly or fully overlap with IgE binding sites on allergens is tempting. However, so far no allergen-specific nanobody fulfilling these criteria was reported indicating that it might be rather difficult to raise allergen-specific nanobodies of sufficient affinities. Whether the current lack of such nanobodies is owed to some inherent structural or functional properties of nanobodies and/or the camelid immune system or the simple reason that the current research focus in the allergy field is on AIT and its improvement has to be resolved. If allergen-specific nanobodies are identified that competitively block allergen binding to IgE and thus abrogate IgE-mediated allergic inflammation, we assume that they will represent appropriate tools for future allergy treatment. Their economic properties, i.e. low production costs encouraged researchers to elaborate antibody engineering of these single-domain antibodies for diverse applications in biotechnology and medicine. This gathered knowledge will facilitate the implementation of modified allergen-specific nanobodies tailored to the needs of allergy treatment. Nanobodies can be easily formatted for a particular application *e.g.*, modified as recognition modules in large constructs or as bi- or oligo-specific, bi- or oligo-valent derivatives.

With the availability of allergen-specific nanobodies or their derivatives with inhibitory potential, it should be possible to examine engineered candidates in proof of concept testings for efficacy and safety in experimental animal models to identify promising nanobody-based drugs for clinically relevant allergens.

## Author Contributions

SF, IZ and ST reviewed the literature, IZ and ST generated figures. SF and ST wrote the manuscript draft. All authors contributed to the article and approved the submitted version.

## Funding

SF was supported by Austria Science Fund (FWF) grants F4607 and I3946-B33 and ST was supported by the Russian Foundation for Basic Research (RFBR) grant 18-515-14003.

## Conflict of Interest

The authors declare that the research was conducted in the absence of any commercial or financial relationships that could be construed as a potential conflict of interest.

## Abbreviations

AIT, Allergen-specific ImmunoTherapy; EBV, Epstein-Barr Virus; Fab, antigen-binding Fragment of antibodies; HCAb, Heavy Chain-only Antibody; HumAb mice, transgenic mice that produce fully human antibodies; PCA, Passive Cutaneous Anaphylaxis; scFv, single chain Fragment variable (recombinant derivative of a classical antibody); V_H_H, Variable domain of Heavy chain of Heavy chain-only antibodies.
